# A Tumor Suppressor Gene, N-myc Downstream-Regulated Gene 1 (NDRG1), in Gliomas and Glioblastomas

**DOI:** 10.3390/brainsci12040473

**Published:** 2022-04-03

**Authors:** Yukiko Nakahara, Hiroshi Ito, Hiroki Namikawa, Takashi Furukawa, Fumitaka Yoshioka, Atsushi Ogata, Jun Masuoka, Tatsuya Abe

**Affiliations:** Department of Neurosurgery, Saga University, Saga 849-8501, Japan; f8257@cc.saga-u.ac.jp (H.I.); st8737@cc.saga-u.ac.jp (H.N.); sm2517@cc.saga-u.ac.jp (T.F.); yoshiokf@cc.saga-u.ac.jp (F.Y.); ogata.a24@gmail.com (A.O.); masuoka@cc.saga-u.ac.jp (J.M.); abet@cc.saga-u.ac.jp (T.A.)

**Keywords:** N-myc downstream-regulated gene 1, glioblastoma, glioblastoma stem-like cells

## Abstract

The development of potent and selective therapeutic approaches to glioblastoma (GBM) requires the identification of molecular pathways that critically regulate the survival and proliferation of GBM. Glioblastoma stem-like cells (GSCs) possess stem-cell-like properties, self-renewal, and differentiation into multiple neural cell lineages. From a clinical point of view, GSCs have been reported to resist radiation and chemotherapy. GSCs are influenced by the microenvironment, especially the hypoxic condition. N-myc downstream-regulated gene 1 (NDRG1) is a tumor suppressor with the potential to suppress the proliferation, invasion, and migration of cancer cells. Previous studies have reported that deregulated expression of NDRG1 affects tumor growth and clinical outcomes of patients with GBM. This literature review aimed to clarify the critical role of NDRG1 in tumorigenesis and acquirement of resistance for anti-GBM therapies, further to discussing the possibility and efficacy of NDRG1 as a novel target of treatment for GBM. The present review was conducted by searching the PubMed and Scopus databases. The search was conducted in February 2022. We review current knowledge on the regulation and signaling of NDRG1 in neuro-oncology. Finally, the role of NDRG1 in GBM and potential clinical applications are discussed.

## 1. Introduction

**Figure 1:**
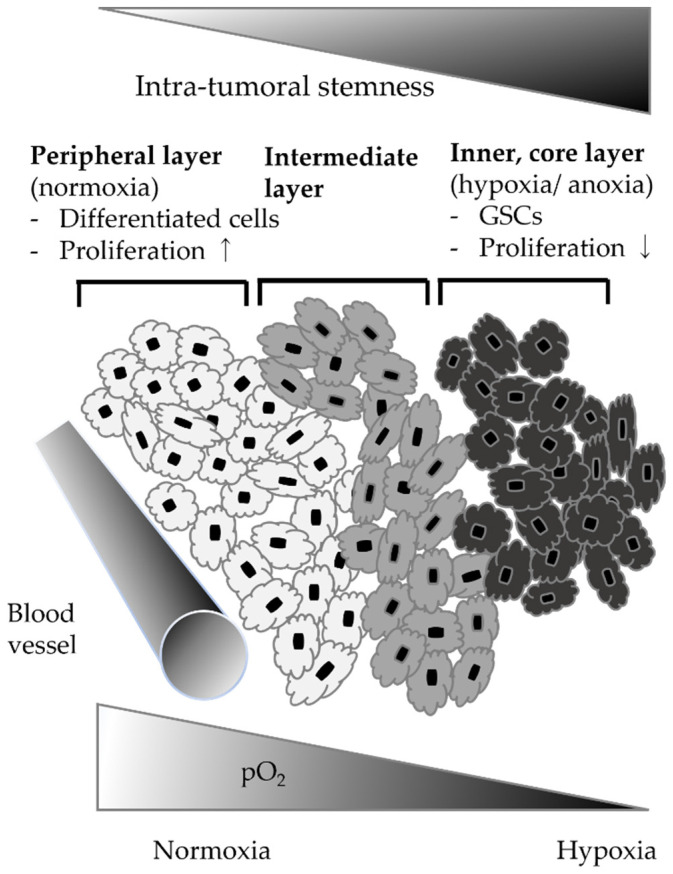
Phenotypic and molecular characteristics of the three layers that comprise the glioblastoma tumor.

Glioblastoma (GBM) is the most malignant primary brain tumor in adults, with a grade IV malignancy according to the World Health Organization (WHO) classification [1]. Although molecular biological studies have provided much insight into the tumorigenesis of GBM, it remains a disease with a poor prognosis, with a median patient survival of 15 months, even with multimodality treatment consisting of surgery, radiation therapy, and chemotherapy [1]. On the clinical side, the presence of glioblastoma stem cells (GSCs) that share the characteristics of normal neural stem cells, i.e., neuroblastogenesis, self-renewal, and differentiation into various neuronal cell lineages, is considered important [2,3,4]. GSCs have been identified as a cause of efficient recurrence of GBM, are resistant to radiotherapy and chemotherapy, and remain after multimodality treatment [5,6,7,8]. In addition, the phenotypic profiles of the GBM cells in association with the oxygen gradient have been investigated. The model was indicated by three layers: an inner highly hypoxic/anoxic layer characterized by GSCs with low proliferation; an intermediate layer—a mildly hypoxic state with immature and proliferating tumor precursor cells; and a peripheral layer—a more vascularized and oxygenated area with differentiated GBM cells [6,9,10] (Figure 1). Different levels of hypoxia in the microenvironment may affect the activation of HIFs, which may be a critical factor in the invasiveness and proliferation activity of GBM [6,9,10,11,12]. Hypoxia-inducible factor-1α (HIF-1α) is known to play an essential role in the regulation of genes induced or upregulated by the hypoxic tumor microenvironment [9,10,11], which in turn are important to the regulation of stemness [5,13,14].

**Figure 2:**
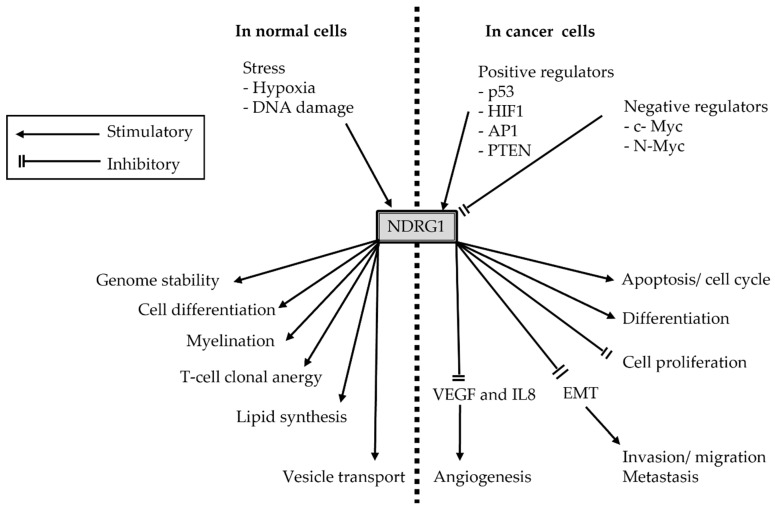
Schematic diagram of the putative functions of NDRG1 in normal or cancer cells. The NDRG1 protein responds to stress and induces genome stability, differentiation, myelination, maintenance of T cell clonal anergy, lipid synthesis, and vesicle transport. In cancer cells, NDRG1 has an inhibitory action on cell proliferation, invasion, migration, metastasis, and angiogenesis, and promotes apoptosis and differentiation. Abbreviations: AP1, activator protein 1; VEGF, vascular endothelial growth factor; IL-8, interleukin-8.

Among the many genes whose expression are regulated by HIF-1α is N-myc downstream-regulated gene 1 (NDRG1). NDRG1 is a member of the NDRG family, and the others are NDRG2, NDRG3, and NDRG4 [15,16]. NDRG1 is strongly expressed in the cerebral cortex; the other members of the NDRG family are also expressed in the spinal cord during development, in which the NDRG family plays a role in the differentiation of the central nervous system [17]. The expression of *NDRG1* mRNA varies among human tissues, with higher expression in the prostate, brain, kidney, placenta, and intestinal tissues [18,19]. NDRG1 is an intracellular protein whose expression is altered under the influence of various stress conditions and molecules [17,19,20,21,22,23]. Although the exact cellular functions of this protein have not been elucidated, mutations in *NDRG1* or aberrant expression of this protein have been associated with tumor-suppressive and oncogenic phenotypes, which collectively suggest that NDRG1 functions in a tissue-specific manner [19,24,25,26,27,28]. NDRG1 is regulated under stress conditions such as starvation or hypoxia. Figure 2 shows the role of its function in normal physiology and its tumorigenesis in cancer cells.

NDRG1 expression is correlated inversely the with survival of GBM patients and is therefore considered a cancer suppressor gene in GBM [29,30,31,32]. Furthermore, NDRG1 is a molecule that is affected under hypoxia, and the protein expression was significantly increased in GBM cells in the hypoxic state [33]. Since the tumor microenvironment, especially hypoxia, is thought to be one of the causes of GBM cells acquiring resistance to therapy, it is important to elucidate the function of this molecule and the regulatory mechanism of its expression. In this review, we examine the literature on NDRG1 in the area of gliomas including GBM cells and GSCs and suggest the potential of novel therapies focusing on NDRG1 for GBM.

## 2. Literature Analysis Methods

The present review was conducted by searching the Scopus and PubMed databases using the key terms “N-myc downstream-regulated gene 1” and “glioma” or “glioblastoma” in the title abstract or keywords. The search was conducted in February 2022. Publicly available, peer-reviewed literature written in English was included and limited to original articles, except reviews and book chapters. The literature was systematically retrieved using the Preferred Reporting Items for Systematic Reviews and Meta-Analyses guidelines; however, relevant data were extracted to help readers’ understanding.

## 3. Results

A total of 26 articles were identified using the search algorithm on Scopus and PubMed. The full manuscript of 21 articles was reviewed and five articles (one proceeding, two review articles, and two book chapters) were excluded due to the type of literature.

### 3.1. NDRG1 Expression in Glioma and GBM

Previous studies investigated the association between the expression of NDRG1 protein in surgical tissue sections using immunohistochemistry and the survival duration of patients with GBM. We demonstrated by Kaplan−Meier analysis that patients with high expression of NDRG1 in specimens of tumor tissue had significantly longer overall survival (OS) than those with low expression of NDRG1 [29]. Furthermore, the rates of positive cells of NDRG1 were positively correlated with the longer survival time in GBM patients [29]. Additionally, a large study in 168 patients with glioma with different WHO grade identified that decreased NDRG1 expression was negatively correlated with WHO grade; moreover, low expression of NDRG1 was associated with significantly lower OS, independent of any prognostic factors [30]. Recently, Yang et al. investigated the polymorphisms of the *NDRG1* gene in 1061 participants, including 558 patients with glioma and 503 healthy individuals, and identified a certain relationship between the polymorphism and the risk of glioma development [31]. Furthermore, experiments using human glioma cell lines found NDRG1 overexpression inhibits cell proliferation and invasion and suppresses tumorigenesis in the subcutaneous tumor mouse model [32]. From these findings, NDRG1 has been considered a potent tumor suppressor in gliomas.

Meanwhile, a study showed that high-grade glioma had the higher expression of *NDRG1* mRNA and protein compared with grade II glioma [20]. Furthermore, when the target was limited to grade II glioma, post-surgically untreated grade II gliomas showed that moderate-to-high expression of the NDRG1 protein was correlated with growth delay and improved progression-free survival, but not OS [34].

Collectively, although these investigations showed that NDRG1 might be suggested to have the potential of a tumor suppressor gene in glioma, several factors might lead to poor prognosis and reduced OS, such as tumor environment (e.g., hypoxia, stress condition) and genotoxic changes by chemotherapy. Furthermore, in the NDRG family, NDRG2 acts in a tumor-suppressive manner similar to NDRG1, while NDRG4 is lowly expressed in gliomas and is considered as a poor prognostic factor. The function of NDRG1 in gliomas may be affected by the balance with other NDRG family genes and requires further study.

### 3.2. NDRG1, Cancer-Related Genes and Pathway

#### 3.2.1. NDRG1 and p53 Associated Apoptosis

NDRG1 shows the biphasic expression through the cell cycle, which has a peak in the G1 and G2/M phases and decreased to the lowest level in the S phase [35]. Overexpression of NDRG1 is known to downregulate the expression of cyclin D1, a Wnt-responsive gene, and to suppress cell cycle progression [36].

Activation of NDRG1 occurs due to the binding of p53 to its promoter [37]. NDRG1 is also a protein located at the centromeres associated with a microtubule corresponding with a p53-dependent spindle checkpoint [18,19]. Furthermore, following DNA damage agents, NDRG1 expression is induced and dependent on p53 [18,37]. In colon cancer cell lines, NDRG1 was required to induce p53-mediated apoptosis [37]. Conversely, studies using lung cancer cell lines showed no association between NDRG1 expression and DNA damage, although p53 was elevated [38]. The regulation of NDRG1 via p53 indicates the potential of cell- and tissue-specific manners. In experiments with glioma cell lines, when NDRG1 overexpression was enforced using retroviral constructs expressing NDRG1, the percentage of apoptotic glioma cells increased relatively higher than in untransfected cells [32]. Similarly, NDRG1 knockdown using small interfering (si)RNA targeting human NDRG1 was shown to decrease glioma cell apoptosis [32,39], following a decrease in cleavage of caspase 3 [39]. Although a high percentage of GBM are known to have mutated p53, there have been no studies on the association between mutant p53 and NDRG1. To develop new NDRG1-mediated GBM therapies, further studies will be required.

#### 3.2.2. NDRG1 and Phosphatase and Tensin Homolog (PTEN), Phosphoinositide 3-Kinase (PI3K)/AKT Pathway

The PTEN/PI3K/AKT signaling pathway is validated to be involved in the development and growth of multiple cancers by many previous studies [40,41,42,43]. The growth of glioma cells is facilitated by inhibition of PTEN, through the regulation of the PI3K/AKT pathway [43,44]. Overexpression of PTEN induces downregulation of PI3K/AKT signaling and reduces the migration in glioma cells [45]. Previous in vitro and in vivo studies of gliomas also showed that the phosphorylation of AKT expression was elevated, and its expression was reversed in correlation with the expression of PTEN [43,44].

The NDRG1 protein regulates the negative feedback loop that links the PI3K/AKT pathway and PTEN [28]. In cancer cells, the balance in the PI3K/PTEN feedback loop is frequently lost, leading to increased PI3K signaling and reduced PTEN levels [28]. NDRG1 knockdown by siNDRG1 in U87 MG showed induction of AKT phosphorylation, suggesting that NDRG1 represses the cell proliferation and invasion through the PI3K/AKT pathway [32]. The study of the microrchidia family CW-type zinc finger 2 (MORC2), which was a chromatin modifier, reported that MORC2 binding to the NDRG1 promotor inactivated PTEN/PI3K/AKT signaling and promoted the growth of glioma cells [41]. Furthermore, glycogen synthase kinase 3ß (GSK3ß), involved in the regulation of cell proliferation, is a key downstream target of the PI3/AKT pathway [46]. Our previous study indicated that NDRG1 and GSK3ß promoted each other’s protein degradation and destabilization and negatively regulated their expressions [29].

#### 3.2.3. NDRG1 and Myc

The overexpression of the N-myc and c-myc binds to the N-myc binding motif close to the initiation promoter and induces the suppression of NDRG1 expression [47,48]. The expression of NDRG1 and Myc was investigated in 168 cases of glioma, and the expression was compared with the WHO grade and survival rate of the patients [30]. NDRG1 expression of mRNA and protein was decreased in gliomas compared with a normal brain. Both mRNA and protein expression of Myc were higher in gliomas, and the expression increased from WHO grade I to WHO grade IV, which was a reverse expression of NDRG1 [30]. The transcriptional repression of human NDRG1 by Myc may be involved in glioma progression.

### 3.3. NDRG1 and Epithelial−Mesenchymal Transition (EMT) in Glioma Invasion

NDRG1 protein-bound in the membrane was discovered mainly alongside the adherent junctions [49]. In cancer, the *NDRG1* gene is considered to engage in the suppression of metastasis to negatively associate with the migration of metastatic cancer cells [26,50,51]. Thus, NDRG1 reduces metastatic potential by the formation of adherent bounds, increased adhesion of the cell to cell, and inhibition of migration and invasion.

**Figure 3:**
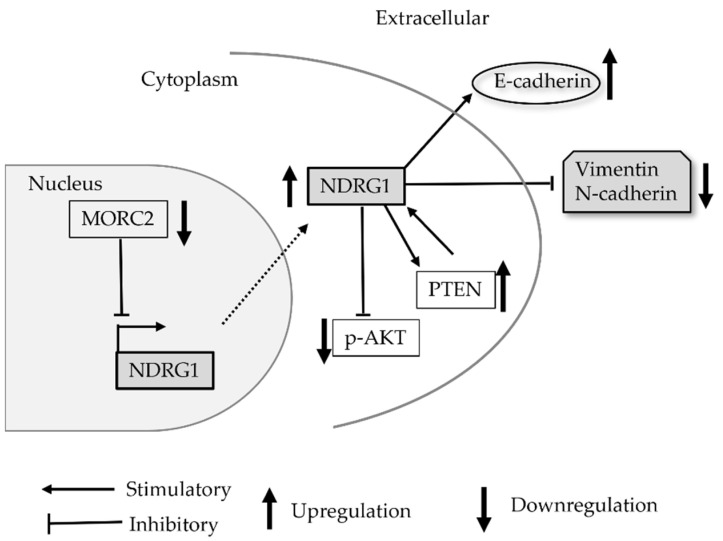
Simplified schematic showing the interaction of NDRG1 with EMT-related genes in references [32,41]. Elevated protein expression of NDRG1 in the cytoplasm is stimulatory to E-cadherin, whereas vimentin and N-cadherin are repressively regulated through the PTEN/AKT pathway in both references. MORC2, which is an oncogene that binds with histone deacetylase 4 and acts as a transcriptional repressor, is studied in reference [41]. Abbreviations: MORC2, microrchidia family CW-type zinc finger 2.

In glioma cells and specimens of surgical resection, EMT-associated proteins, including vimentin, N-cadherin, and E-cadherin as an invasive marker, have been known to show a marked elevation of expression [52]. Ma et al. studied the expression of vimentin, N-cadherin, and E-cadherin in NDRG1 overexpressed U87MG using retroviral constructs and in the knockdown expression of NDRG1 using siNDRG1 [32]. Negative associations were shown between vimentin, N-cadherin, and NDRG1 expression; moreover, E-cadherin showed a positive correlation with NDRG1 expression. This result suggests the possibility that NDRG1 overexpression inhibits glioma cells invasion by modulating vimentin, N-cadherin, and E-cadherin [32]. Furthermore, the results of in vivo experiments such as in tumorous tissue of U251 tumor-bearing mice also showed that the acquisition of NDRG1 function repressed expression of EMT-related proteins and inhibited the development of tumor, proliferation, and migration (Figure 3) [41]. These results have indicated that NDRG1 may play a significant role in regulating the growth and invasion of glioma cells.

### 3.4. NDRG1 in Stress Conditions

#### 3.4.1. A Role of NDRG1 as the Substrate of Serum/Glucocorticoid-Regulated Kinase 1 (SGK1)

NDRG1 plays a role in physiological stress conditions. NDRG1 is a substrate of serum/glucocorticoid-regulated kinase 1 (SGK1) activated by corticosterone in plasma [53]. According to an elevation of corticosterone in plasma, SGK1 expression and activation increase, specifically in oligodendrocytes, results in causing increased phosphorylation of NDRG1 and leads to various effects for the molecules in downstream including increased expression of adhesion molecules. SGK1 has physiological functions such as regulation of ion channels, differentiation and proliferation of cells, and apoptosis. In GBM, the expression of SGK1 increases significantly [21]. The overexpression of activated SGK1 and phosphorylated NDRG1 implicated the morphologic alterations that were the result of the pathway of SGK1 and NDRG1 induced by stress conditions [22,53].

#### 3.4.2. NDRG1 in GBM Cells under Hypoxic Condition

Considering the microenvironment within the solid tumor, hypoxia is of great importance [20,33,54]. Tumor cells adapt to hypoxic environments and proliferate by activating genes that encode proteins necessary for survival in these environments [6,55,56]. A common survival strategy in response to hypoxia is Pasteur-effect remission, in which HIFs bind to hypoxia-responsive genes [57], which causes them to express proteins that are favorable for survival [10]. Transcription factor HIF-1α accumulates under hypoxic conditions and induces the expression of NDRG1 as one of the hypoxia-inducible genes [6,20]. The NDRG1 gene has three HIF-1 binding sites, one in its promoter and the remaining two in its 3′ untranslated region [58], of which NDRG1 is possibly regulated by HIF-1 through its binding sites in the untranslated region [23]. In hypoxia, NDRG1 regulation is associated with HIF-1α-dependent and HIF-1α-independent pathways [23].

Since hypoxia is a very important environmental factor in GBM research, several studies on alterations in NDRG1 expression in GBM cells under hypoxic conditions reported significant upregulation of mRNA and/or protein NDRG1 expression [11,20,21,33,59,60]. The molecular and phenotypic characteristics of tumors are characterized by three layers according to a hypoxic concentric gradient: the inner core layer, the middle layer, and the peripheral layer (Figure 1). The invasiveness and proliferation activity of GBM are severely affected by differences in oxygen levels in the microenvironment that alter the activation of HIFs [6,20,33,55]. Under prolonged moderate (1% O_2_) to severe (0.1% O_2_) hypoxia, mRNA and protein NDRG1 expression generally increased and were regulated in an oxygen-dependent manner [33]. In GBM cells, NDRG1 protein was also highly stable, cell-specific, and dependent on the oxygen concentration [20]. The increased expression of the NDRG1 protein in GBM cell lines was time-dependent during hypoxia [11,33,60]. HIF-1α has been found to upregulate not only NDRG1 but also carbonic anhydrase IX (CA-IX), which is a transmembrane N-glycosylated isoenzyme. Said et al. showed that NDRG1 was also expressed in the acute phase of hypoxia, while CA-IX was expressed during the more chronic phase [60]. Furthermore, *NDRG1* mRNA expression was downregulated, while its protein did not show downregulation after reoxygenation [33]. This result suggested posttranscriptional regulation of NDRG1 expression.

Together with HIFs, early growth response factor 1 (Egr-1), which is a transcription factor activated by hypoxia, plays a major role in the survival of tumor cells under hypoxic conditions [61]. Egr-1 is thought to activate and positively regulate NDRG1 in a variety of cancers; however, in GBM, Egr-1 was not involved in the regulation of NDRG1 expression, indicating that HIF might be the main regulator [59].

Furthermore, induction of NDRG1 expression via HIF1 under hypoxia requires an increase in intracellular calcium (Ca^2+^) [19,62]. Intracellular Ca^2+^ elevation is not directly related to induction of HIF-1α and promotion of HIF-1α-dependent transcription [19,62]. Intracellular Ca^2+^ upregulation may affect NDRG1 induction through signaling pathways or transcriptional mechanisms, differing from HIF-1 [19,62]. Thus, the role of HIF-1α and the increase of intracellular Ca^2+^ in regulation of NDRG1 is undetermined to date. At this time, there are no studies on GBM regarding the relationship between elevation of intracellular Ca^2+^ and NDRG1 expression, and future studies are needed.

In this way, the regulation of NDRG1 under hypoxia is highly complex, and its cellular function is still a matter of debate.

### 3.5. Influence of NDRG1 on Resistance to GBM Treatment

#### 3.5.1. O^6^-Methylguanine-DNA Methyltransferase (MGMT)

MGMT is a key enzyme responsible for the resistance of GBM to the alkylating agent temozolomide (TMZ) [7,21,55,63]. Weiler et al. demonstrated that NDRG1 increases the protein stability and promotes the activity of MGMT and that NDRG1 is a predictive marker for responsiveness to chemotherapy with alkylating agents such as TMZ [21]. In addition, NDRG1 is affected by not only the tumor microenvironment but also glioma treatments such as radiation, chemotherapeutic agents, and steroids, and its pathway is the mechanistic target of the rapamycin C2 (mTORC2)/SGK1 pathway [21]. From the point of view of this report, NDRG1 may be a promising target for GBM therapy because NDRG1 expression and function are affected by conventional GBM therapies, if methods will be developed to reduce these effects in the future.

#### 3.5.2. Glioblastoma Stem-like Cells (GSCs)

The association with the microenvironment of GSCs has been studied, including perivascular niches [12], periarteriolar niches [47,64], peri-hypoxic niches [65], peri-immune niches [66], and extracellular matrix niches [6,55]. The hypoxic environment has induced the expression of GSCs markers, including the sex-determining region Y-box2 (SOX2), octamer-binding transcription factor 4 (OCT4), and CD133, resulting in the dedifferentiation of GBM cells into GSCs [6,9,55,56,67]. HIF-1α is known to play an essential role in the regulating stemness of GSCs [5,13,14]. HIF-1α was found to have a more general function in the maintenance of GSCs [6,55,56], whereas, in contrast to HIF-1α, HIF-2α directly promoted the phenotype of GSCs upregulating the expression of GSCs markers [6,9].

According to genome-wide expression and methylation studies, GBM has classified three types: proneural (PN), mesenchymal (MES), and classical. Each type of GSC is known to show a different phenotype. In general, GSCs of MES are more aggressive and invasive and have a poorer prognosis than GSCs of PN [68]. Few articles investigated the role of NDRG1 in GSCs. The study of NDRG1 in GSCs investigated its expression by classifying GBM subgroups [69]. In GSCs of PN, the decreased expression of NDRG1 suppressed self-renewal, promoted differentiation, and significantly inhibited tumorigenesis. Conversely, NDRG1 overexpression in GSCs induced the transition from PN to MES and improved the highly malignant phenotype [69]. The results also indicated that the transition from PN to MES was regulated by the balance between the expression of both NDRG1 and achaete-scute homolog 1 (ASCL1), which is a specific gene of PN type of GBM and has a relevant role in the neuronal differentiation of GSCs [69].

It is important to define the role of NDRG1 and its regulatory mechanisms in GSCs, which are one of the causes of GBM resistance to multidisciplinary therapy. Further studies are needed to determine whether the microenvironment affects GSCs and prevents effective treatments to eradicate GSCs, as the interactions between GSCs and their niches have not been clarified [2,3,4,34].

### 3.6. Development of Anti-GBM Therapy Associated with NDRG1

**Figure 4:**
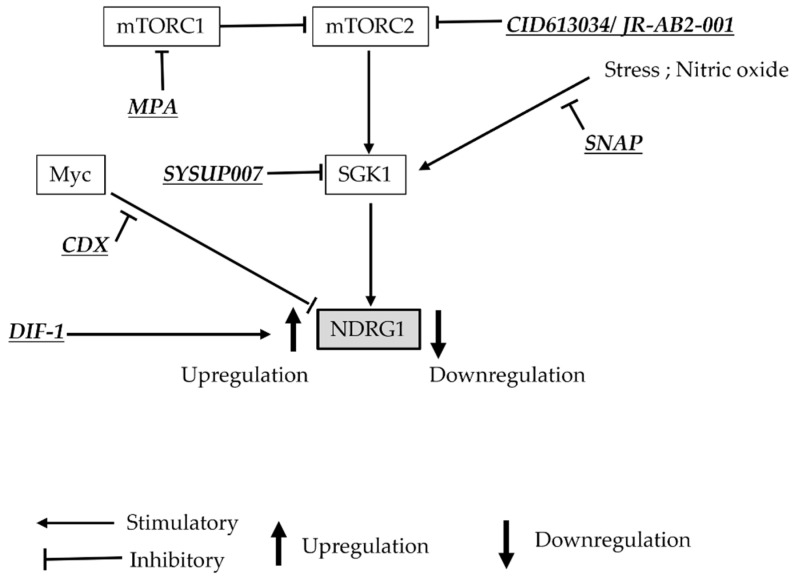
A simplified diagram of the sites associated with NDRG1 of agents reported as candidates for GBM treatment and changes in NDRG1 expression.

The establishment of effective and selective novel therapies for GBM with the poorest prognosis requires the investigation of regulating mechanisms of various gene expressions, functions of proteins, and molecular pathways that critically control the survival and growth of GBM cells. In addition, the microenvironment surrounding tumors and GSCs, which are thought to contribute to recurrence, must be studied in detail in the development of therapies for GBM. Various agents already used as treatments for other diseases have been investigated on their effect of suppression in GBM cells. Table 1 shows the characteristics of each agent and the consequences of NDRG1. In studies of these agents, it has been considered that NDRG1 could be the target of an agent, alter its expression, and be involved in mechanisms of efficiency in GBM cells [11,29,70,71,72,73,74]. Figure 4 shows the relationship between agents reported previously and NDRG1.

Three articles investigated and showed the antitumor effect of each agent in vivo study using xenografts [29,70,75]. Interestingly, Tai et al. demonstrated that the nitric oxide donor, S-nitroso-N-acetylpenicillamine, acted principally through posttranslational modification of p53, phosphorylated NDRG1, and MGMT protein stability in TMZ resistant GBM cells [75]. Suppressions of tumor growth and proliferation in GBM cells and sphere formation of GSCs were observed in vitro and in vivo after being treated with differentiation-inducing factor-1 (DIF-1), which affects the Wnt/ß-catenin signaling pathway [29,46,76,77]. This study also demonstrates a novel mechanism of DIF-1. The growth suppression by DIF-1 was significantly augmented by repressed NDRG1, followed by regaining of GSK3ß and phosphorylated expression of AKT, indicating that upregulation of NDRG1 by DIF-1 has implied the direct suppressio n of cell growth [29]. Based on these findings, NDRG1 may contribute as a novel therapeutic targeting molecule in the treatment of GBM.

## 4. Conclusions

The present review summarized that NDRG1 plays a critical role in progression, differentiation, and invasion in glioma and GBM, through its interaction with various key molecules and signaling pathways. GBM is a heterogeneous tumor, and its cells adapt to hypoxia and other microenvironments and alter various genetic abnormalities and signaling pathways to survive against multimodal therapies. GSCs that are capable of self-renewal and are resistant to conventional radiotherapy and chemotherapy, also existing within the GBM tumor mass, form the heterogeneity of GBM. In addition, NDRG1 also has a variety of physiological effects, and its expression is regulated by a wide range of mechanisms. From the point of view of the heterogeneity of GBM tumors themselves and the diversity of NDRG1 functions, it is not easy to study NDRG1 in GBM; however, some kind of breakthroughs in NDRG1 research is expected to lead to the development of novel therapies rapidly. In conclusion, further elucidation of the molecular mechanisms that underlie the anti-GBM effects of NDRG1 will facilitate the development of new therapies that reduce resistance to radiation and chemotherapy and the stemness of GSCs.

## Figures and Tables

**Table 1:** Studies of novel anti-GBM therapies.

Agent	Structure and Function	Year of Publication	Effect on Tumor	Alteration of NDRG1	Ref.
SYSUP007	Combined rhein with the HDAC inhibitor (SAHA)	2020	Inhibition of proliferation, invasion, and migration (in vitro)	Upregulated expression	[74]
DIF-1	One of the effector molecules inhibiting growth and promoting differentiation	2019	Direct mediation of cell growth suppression (in vitro and in vivo)	Upregulated expression	[29]
SNAP	Nitric oxide donor	2019	As combined with TMZ, inhibition of tumor growth in TMZ resistant GBM (in vitro and in vivo)	Downregulated phosphorylation	[75]
CID613034	mTOR2 inhibitor	2017	Inhibition of proliferation and invasion (in vitro)	Downregulated phosphorylation	[70]
JR-AB2-011	(CID613034 analog)		Reduction in tumor proliferation (in vivo)		
CDX	A three-finger neurotoxin purified from the venom of the Malayan krait (Bungarus candidus)	2012	Inhibition of cell proliferation and promoting apoptosis (in vitro)	Upregulated expression	[72]
MPA	Inhibitor of IMPDH	2008	Antiangiogenic and antifibrotic activity(in vitro)	Upregulated expression	[71]

Abbreviations: HDAC, histone deacetylase; SAHA, suberoylanilide hydroxamic acid; DIF-1, differentiation-inducing factor-1; SNAP, S-nitroso-N-acetylpenicillamine; CDX, candoxin; MPA; mycophenolic acid; IMPDH, inosine monophosphate dehydrogenase.

## Data Availability

Not applicable.

## Abbreviations

GBM	Glioblastoma
GSCs	Glioblastoma stem-like cells
NDRG1	N-myc downstream-regulated gene 1
HIF	Hypoxia-induced factor
WHO	World Health Organization
OS	Overall survival
AP1	Activator protein 1
VEGF	Vascular endothelial growth factor
IL-8	Interleukin-8
(s.i.)RNA	Small interfering ribonucleic acid
PTEN	Phosphatase and tensin homolog
PI3K	Phosphoinositide 3-kinase
MORC2	Microrchidia family CW-type zinc finger2
GSK3ß	Glycogen synthase kinase
EMT	Epithelial−mesenchymal transition
SGK	Substrate of serum/glucocorticoid-regulated kinase 1
CA-IX	Carbonic anhydrase IX
Egr-1	early growth response factor 1
MGMT	O^6^-methylguanine-DNA methyltransferase
TMZ	Temozolomide
mTOR	Mechanistic target of rapamycin
SOX2	Sex determing regionY-box2
OCT4	Octamer-binding transcription factor 4
PN	Proneural type
MES	Mesenchymal type
ASCL1	Achaete-scute homolog 1
DIF	Differentiation-inducing factor-1
HDAC	Histone deacetylase
SAHA	Suberoylanilide hydroxamic acid
SNAP	S-nitroso-N-acetylpenicillamine
CDX	Candoxin
MPA	Mycophenolic acid
IMPDH	Inosine monophosphate dehydrogenase
